# Unusual Manifestation of Ulcerative Colitis

**DOI:** 10.1155/2019/5163213

**Published:** 2019-01-31

**Authors:** Amna Basheer M. Ahmed, Badr M. Rasheed Alsaleem

**Affiliations:** Pediatric Gastroenterology Division, Children's Hospital, King Fahad Medical City, Riyadh, Saudi Arabia

## Abstract

The relationship of inflammatory bowel disease (IBD) and chronic recurrent multifocal osteomyelitis (CRMO) is understood as extraintestinal rheumatic manifestations. CRMO is a chronic, relapsing, inflammatory, noninfectious disorder of the skeletal system of unknown origin. The disease course is not always recurrent. The association of CRMO and ulcerative colitis (UC) is very rarely reported. We report a case of a 10-year-old Saudi female who was diagnosed with CRMO, when she developed fever in association with left foot pain, and ulcerative colitis was confirmed endoscopically and histologically based on a previous settled diarrheal illness and severe iron deficiency anemia which required blood. Both conditions responded well to IBD therapy. To the best of our knowledge, this is the first reported case of chronic, multifocal osteomyelitis associated with pediatric UC in Saudi Arabia. This report supports the use of IBD therapy in treating CRMO.

## 1. Introduction

Chronic recurrent multifocal osteomyelitis (CRMO) is a rare condition, characterized by recurrent episodes of bone inflammation mimicking acute osteomyelitis. It is a self-limited relapsing noninfectious inflammatory condition usually affecting children and adolescents. CRMO was first described in 1972 (Giedion et al.) [1]. CRMO resembles the most severe form of chronic nonbacterial osteomyelitis (CNO) [2]. Some children affected by chronic nonbacterial osteomyelitis (CNO) do not have multiple lesions or a recurrent course [3, 4]. Between 200 and 300 cases of CRMO have been reported in the literature worldwide, consisting mainly of case series with relatively brief follow-up durations [5].

The relationship between CRMO and inflammatory bowel disease is poorly described, and CRMO is considered to be an extraintestinal manifestation of IBD [1, 6]. Ulcerative colitis (UC) and chronic recurrent multifocal osteomyelitis (CRMO) association is less frequently reported compared to Crohn's disease (CD). Kim et al. reported the first case of chronic recurrent multifocal osteomyelitis of the sternocostal region, associated with UC in an adult patient [7].

## 2. Case Report

**Figure 1:**
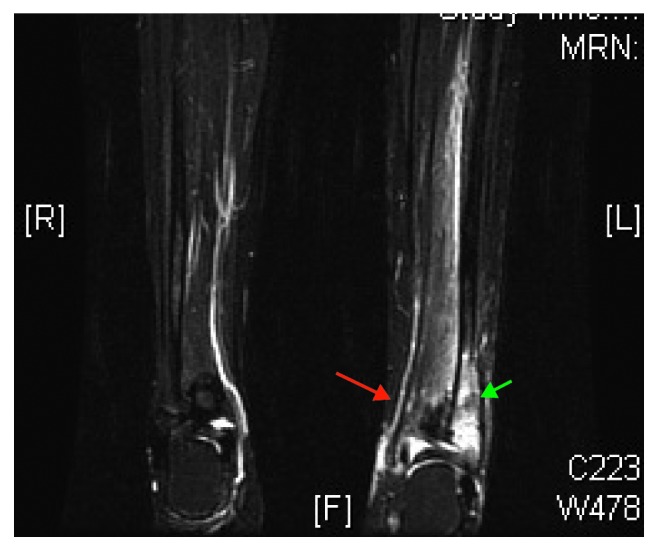
MRI of distal left fibula (green arrow) and tibia (red arrow) showing multifocal chronic osteomyelitis.

An 11-year-old Saudi female, presented with fever, pain, and swelling in the left foot for a 6-month duration. Magnetic resonant imaging (MRI) of the left lower limb confirmed the diagnosis of multifocal, chronic osteomyelitis involving the distal left fibula, lower part of both tibiae, and metatarsal bones of both feet (Figure 1). Surgical incision and drainage in the affected lower limb was done, and the aspirated fluid and bony tissue biopsy excluded fungal and bacterial causes of chronic osteomyelitis, including mycobacterial tuberculosis. Initial management by intravenous antibiotic therapy was provided; however, it was stopped following negative bacterial culture.

Subsequently, she was referred to our service because of stunted growth, past history of chronic bloody diarrhea, abdominal pain, and multiple blood transfusions following recurrent, and severe hemoglobin drop. Family history was remarkable for Crohn's disease in a paternal aunt.

Physical examination revealed pallor and stunted growth.

**Figure 2:**
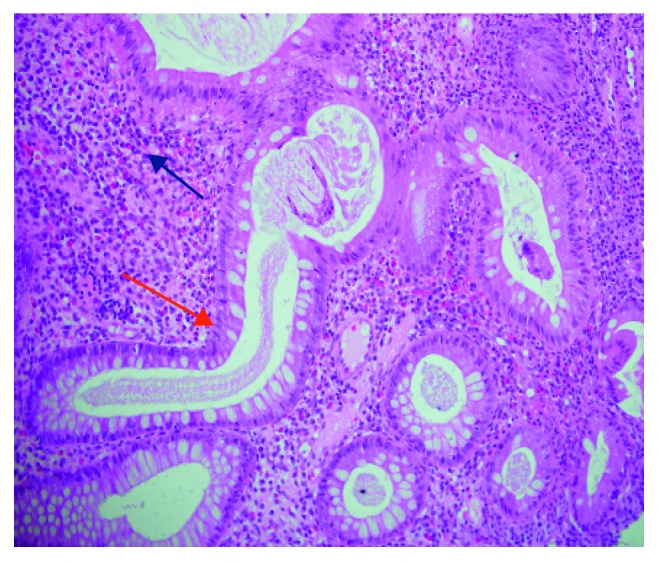
Histopathology of descending colon biopsy, showing UC picture: distorted crypts (red arrow) with lamina propria chronic mononuclear inflammatory cells infiltrate (blue arrow) (H&E stain, ×40).

Initial laboratory workup showed features of iron deficiency anemia, high erythrocyte sedimentation rate (ESR), and high C-reactive protein (CRP). Upper digestive endoscopy was unremarkable, with normal duodenal, stomach, and esophageal histology. The diagnosis of UC was established following clinical, colonoscopic, histological, and radiological findings. Hence, colonoscopy showed features of pancolitis with pseudopolyps in the entire colon; histology showed features of chronic active colitis, crypt architectural distortion, and absence of granuloma (Figure 2); terminal ileum (TI) was normal macroscopically and histologically; and magnetic resonance enterography (MRE) excluded small bowel disease. The final diagnosis of ulcerative colitis UC in association with chronic multifocal osteomyelitis was made. A remarkable remission for intestinal and bony symptoms as well was achieved following IBD therapy prednisolone, mesalamine, and azathioprine.

## 3. Discussion

The case describes an 11-year-old girl with settled chronic bloody diarrhea, who presented with a nonrecurrent chronic osteomyelitis. After appropriate tests, UC in association with chronic multifocal osteomyelitis was made. Twenty-seven cases of CRMO in association with IBD had been reported worldwide, and the majority of the affected patients were children [8]. UC symptoms in our patient predated the osteomyelitis symptoms; this is dissimilar to previous reports in which the onset of the bony lesions in the majority of cases preceded that of bowel symptoms by as much as five years [1]. However, in some case reports, IBD symptoms either preceded or occurred simultaneously with those of CRMO. Seven out of 27 reported cases of CRMO in association with IBD had UC and the rest had Crohn's disease (CD) [8].

CRMO is characterized by multifocal nonpyogenic inflammatory bone lesions, a course of exacerbations and remissions, and an association with other inflammatory disorders [9]. Synovitis, acne, pustulosis, hyperostosis, and osteitis (SAPHO) syndrome appears to be a condition related to CRMO and may represent its adult form. The aetiology of CRMO is unknown, but an autoimmune process is implicated, with predisposing genetic factors [10]. CRMO may be a rare extraintestinal manifestation of inflammatory bowel disease; alternatively, these two conditions may be linked by a common genetic susceptibility [11]. Bone inflammation in CRMO is a result of an immune response directed against bone. Numerous data suggest that IBD is also caused by an activated immune system; therefore, cytokines, particularly IL-1, IL-6 and tumour necrosis factor-a (TNF-a), which are released by the inflamed bowel could potentially mediate joint and bony inflammation [1].

The radiographic appearance of CRMO consists of osteolytic lesions with surrounding sclerosis. MRI is more sensitive than plain radiography for assessing the extent and activity of the disease. Bone biopsy is sometimes required to exclude an infectious aetiology or a neoplastic lesion [12].

Jansson et al. proposed four major criteria and six minor criteria for CRMO. Diagnosis is confirmed by the presence of two major or one major and three minor criteria [13].

Chronic recurrent multifocal osteomyelitis (CRMO) was diagnosed in our patient based on two of the three major criteria MRI findings and multifocal bone lesions.

First-line treatment of CRMO is with nonsteroidal anti-inflammatory drugs (NSAIDs), which are effective in the majority of patients [12]. When CRMO occurs in association with inflammatory bowel disease (IBD), nonsteroidal anti-inflammatory drugs (NSAIDs) are relatively contraindicated because of their recognised association with colitis flare. Other treatments used include corticosteroids, methotrexate, sulfasalazine, colchicine, and azithromycin [14].

There are no comparative or randomised trials regarding the choice of therapy. Success with antitumour necrosis factor (TNF) agents and bisphosphonates has been reported recently (reviewed by Ferguson and Sandu [10]).

In our patient, the bony symptoms remarkably improved, without relapse, after using aminosalicylates, corticosteroids, and immunomodulators.

The prognosis of CRMO is generally good,however,a number of patients had persistent disease and therefore remain at risk of physical and psychological compilications [15].

## 4. Conclusions

To the best of our knowledge, this is the first reported case of chronic, nonrecurrent, multifocal osteomyelitis, associated with pediatric UC in Saudi Arabia.

Our case report further supported the efficacy of IBD therapy in treating CRMO.

Since IBD and CRMO association is rare and poorly understood, etiopathology and the natural course of the condition should be done by conducting a multicenter study.
